# Impact of image compression on deep learning-based mammogram classification

**DOI:** 10.1038/s41598-021-86726-w

**Published:** 2021-04-12

**Authors:** Yong-Yeon Jo, Young Sang Choi, Hyun Woo Park, Jae Hyeok Lee, Hyojung Jung, Hyo-Eun Kim, Kyounglan Ko, Chan Wha Lee, Hyo Soung Cha, Yul Hwangbo

**Affiliations:** 1 Healthcare AI Team, National Cancer Center, 323 Ilsan-ro, Ilsandong-gu, Goyang-si, Gyeonggi-do 10408 Republic of Korea; 2 Lunit Inc., 27, Teheran-ro 2-gil, Gangnam-gu, Seoul, 06241 Republic of Korea; 3 Department of Radiology, National Cancer Center, 323 Ilsan-ro, Ilsandong-gu, Goyang-si, Gyeonggi-do 10408 Republic of Korea

**Keywords:** Medical imaging, Radiography

## Abstract

Image compression is used in several clinical organizations to help address the overhead associated with medical imaging. These methods reduce file size by using a compact representation of the original image. This study aimed to analyze the impact of image compression on the performance of deep learning-based models in classifying mammograms as “malignant”—cases that lead to a cancer diagnosis and treatment—or “normal” and “benign,” non-malignant cases that do not require immediate medical intervention. In this retrospective study, 9111 unique mammograms–5672 normal, 1686 benign, and 1754 malignant cases were collected from the National Cancer Center in the Republic of Korea. Image compression was applied to mammograms with compression ratios (CRs) ranging from 15 to 11 K. Convolutional neural networks (CNNs) with three convolutional layers and three fully-connected layers were trained using these images to classify a mammogram as malignant or not malignant across a range of CRs using five-fold cross-validation. Models trained on images with maximum CRs of 5 K had an average area under the receiver operating characteristic curve (AUROC) of 0.87 and area under the precision-recall curve (AUPRC) of 0.75 across the five folds and compression ratios. For images compressed with CRs of 10 K and 11 K, model performance decreased (average 0.79 in AUROC and 0.49 in AUPRC). Upon generating saliency maps that visualize the areas each model views as significant for prediction, models trained on less compressed (CR <  = 5 K) images had maps encapsulating a radiologist’s label, while models trained on images with higher amounts of compression had maps that missed the ground truth completely. In addition, base ResNet18 models pre-trained on ImageNet and trained using compressed mammograms did not show performance improvements over our CNN model, with AUROC and AUPRC values ranging from 0.77 to 0.87 and 0.52 to 0.71 respectively when trained and tested on images with maximum CRs of 5 K. This paper finds that while training models on images with increased the robustness of the models when tested on compressed data, moderate image compression did not substantially impact the classification performance of DL-based models.

## Introduction

The extensive use of medical imaging has led to a rapid increase in the amount of clinical imaging data being created and stored globally^1,2^. To help address the associated storage overhead, the digital imaging and communications in medicine (DICOM) standard allows clinical organizations to reduce file size through image compression^3–5^. Radiological societies in several countries have published recommendations on acceptable compression ratios (CRs) for multiple medical imaging modalities. For example, in radiography, computed tomography, and magnetic resonance imaging, CR recommendations fall in the range of 3 to 50^6^. Most previous studies on the impact of image compression focused on qualitatively evaluating CR thresholds for visually and diagnostically lossless compression when viewed solely by radiologists^7–9^.

The utilization of both machine learning and deep learning (DL) in medical imaging is expansive and continues to grow, with tasks such as classification^10,11^, abnormality detection^12,13^, and risk prediction^14,15^. However, despite the diversity in data storage policies across different hospitals and countries, as well as the fragility of some models when tested on new data^16^, previous studies have overlooked image compression as a factor in model performance. To the best of our knowledge, this is the first work to investigate the impact of image compression on deep learning models in radiology; consequently, we were unable to compare our work to previous results in the literature.

## Results

### Compressed mammograms using different compression ratios

In this study, we used various CRs based on previous literature on image compression and mammograms, including the recommended values from radiological societies in the United Kingdom, Germany, and Canada of 15, 20, and 25 respectively^6^. Additionally, we applied more extreme CRs of 50, 100, 500, 1 K, 5 K, 10 K, and 11 K.

**Figure 1:**
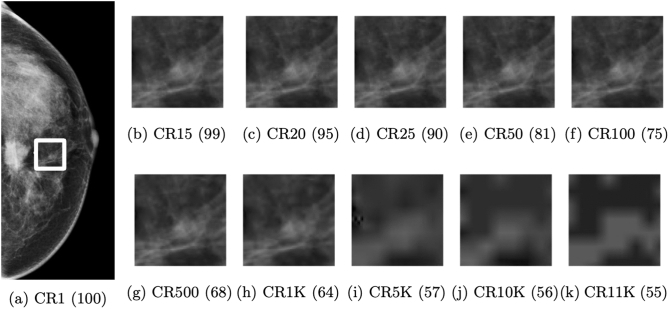
Images with different compression ratios (CRs). Subfigures (**b**)∼(**k**) are the same patched image corresponding to the area demarcated with a white rectangle of in the original image (i.e. subfigure (**a**)) compressed using different CRs. The caption of each subfigure includes both the CR and peak signal-to-noise ratio (PNSR). For example, the patched image tagged with the caption CR1K (64) has a CR of 1 K and PNSR of 64 dB.

Figure 1 presents examples of images compressed with a range of CRs, on a patch extracted from a sample mammogram. Compression was applied via Python using the Glymur package (version 0.9), a binding for the JPEG 2000 reference software OpenJPEG (version 2.3). Each subfigure shows a patch and its respective CR. The blurriness of an image scales with an increase in CR as the amount of compression applied to the patch increases. In addition to the respective compression ratio, we notated the peak signal-to-noise ratio (PSNR) to sub caption of each patch in parentheses. PSNR is a commonly used metric for assessing the quality of a reconstructed image and quantifies the difference between the original and compressed representations on a pixel-by-pixel level^8^. Equation 1 defines PSNR, measured in decibels (dB), for a 16-bit source image $I$ with dimensions $n \times n$ and its $n \times n$ compressed representation $R$:

1 $$PSNR\left( {I,R} \right) = 10 \cdot \log_{10} \left( {\frac{{MAX_{I}^{2} }}{{MSE\left( {I,R} \right)}}} \right) = 10 \cdot \log_{10} \left( {\frac{{\left( {2^{16} - 1} \right)^{2} }}{{\frac{1}{{n^{2} }}\mathop \sum \nolimits_{i = 0}^{n - 1} \mathop \sum \nolimits_{j = 0}^{n - 1} \left( {I\left( {i,j} \right) - R\left( {i,j} \right)} \right)^{2} }}} \right)$$

Equation 1 PSNR for a 16-bit $n \times n$ source image $I$ with and its $n \times n$ compressed representation $R$.

where is the bit-depth of the image $I$ and its compressed representation $R$, and $MSE\left( {I,R} \right)$ is the mean-squared error of the compressed representation. A perfect reconstruction of the original image will have a PSNR of infinity; however, we added a label of 100 to reflect the value outputted by our software implementation.

### Performance evaluation of models using different compression ratios

**Figure 2:**
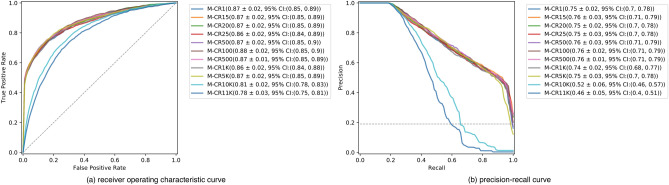
Classification performance of models tested on the compressed images. Subfigure (**a**) is the receiver operating characteristic curve (ROC), while subfigure (**b**) is the precision-recall (PR) curve. Each label in the legend annotates a model trained and tested on images with a single compression ratio, and the performance for area under ROC or PR curves (AUROC or AUPRC) with 95% confidence interval in parentheses.

We first trained three-layer convolutional neural network (CNN3) models on images compressed with a single CR and then evaluated each model on the test images with the same CR. The results are shown in Fig. 2. For both the ROC and PR, the individual curves correspond to the performance, standard deviation, and 95% confidence interval (CI) from five-fold cross validation by model. A model trained on images with a certain CR is denominated as M-CR#. We trained our models using images compressed with CRs between 1 and 5 K (i.e., M-CR1–M-CR5K), which exhibited similar area under ROC curve (AUROC) and area under PR curve (AUPRC), 0.86–0.88 (± 0.01–0.02, 95% CI 0.85 to 0.89) and 0.74–0.76 (± 0.02–0.04, 95% CI 0.68 to 0.79), respectively. In contrast, M-CR10K and M-CR11K perform relatively worse than the others, with AUROC of 0.81 (± 0.02, 95% CI 0.78 to 0.83) and 0.78 (± 0.03, 95% CI 0.75 to 0.81) and AUPRC of 0.52 (± 0.06, 95% CI 0.46 to 0.57) and 0.46 (± 0.05, 95% CI 0.40 to 0.51), respectively.

In addition to our CNN3 models, we performed five-fold cross-validation using ResNet18^17^ models pretrained on ImageNet to investigate the impact of image compression on models with more layers and initialized by pretraining. We chose the ResNet architecture because of its success in previous work on mammogram classification^14,16^. Each model was trained and tested on images compressed with a single CR as in the CNN3 experiment. Compared to the CNN3 model, the ResNet models had worse performance, with AUROC and AUPRC values ranging from 0.70–0.84 to 0.33–0.66 respectively for CR values between 1 and 5 K shown as Table 1. Similar to the CNN3 results, ResNet models trained and tested on images with CRs of 10 K and 11 K saw a performance drop with AUROC values of 0.73 and 0.70 and AUPRC values of 0.38 and 0.33 respectively.

**Table 1:** Classification performance of our three-layer convolutional neural network (CNN3) and pretrained ResNet18 models when tested and trained on different compression ratios.

Model/Data	CR1	CR15	CR20	CR25	CR50	CR100	CR500	CR1K	CR5K	CR10K	CR11K
**(a) AUROC with 95% confidence interval**											
CNN3	0.87 (0.85, 0.89)	0.87 (0.85, 0.89)	0.87 (0.85, 0.89)	0.86 (0.84, 0.89)	0.87 (0.85, 0.90)	0.88 (0.85, 0.90)	0.87 (0.85, 0.89)	0.86 (0.84, 0.88)	0.87 (0.85, 0.89)	0.81 (0.78, 0.83)	0.78 (0.75, 0.81)
ResNet18	0.80 (0.78, 0.83)	0.84 (0.82, 0.87)	0.80 (0.77, 0.83)	0.82 (0.79, 0.84)	0.83 (0.80, 0.85)	0.81 (0.78, 0.83)	0.81 (0.78, 0.84)	0.82 (0.79, 0.85)	0.81 (0.79, 0.84)	0.73 (0.70, 0.76)	0.70 (0.67, 0.73)
**(b) AUPRC with 95% confidence interval**											
CNN3	0.75 (0.70, 0.78)	0.76 (0.71, 0.79)	0.75 (0.70, 0.78)	0.75 (0.70, 0.78)	0.76 (0.71, 0.79)	0.76 (0.71, 0.79)	0.76 (0.71, 0.79)	0.74 (0.68, 0.77)	0.75 (0.70, 0.78)	0.52 (0.46, 0.57)	0.46 (0.40, 0.51)
ResNet18	0.56 (0.53, 0.63)	0.66 (0.61, 0.71)	0.59 (0.53, 0.64)	0.60 (0.54, 0.65)	0.61 (0.56, 0.67)	0.58 (0.52, 0.63)	0.60 (0.55, 0.65)	0.63 (0.58, 0.67)	0.60 (0.55, 0.65)	0.38 (0.33, 0.43)	0.33 (0.29, 0.37)

All reported metrics are from five-fold cross validation.

**Figure 3:**
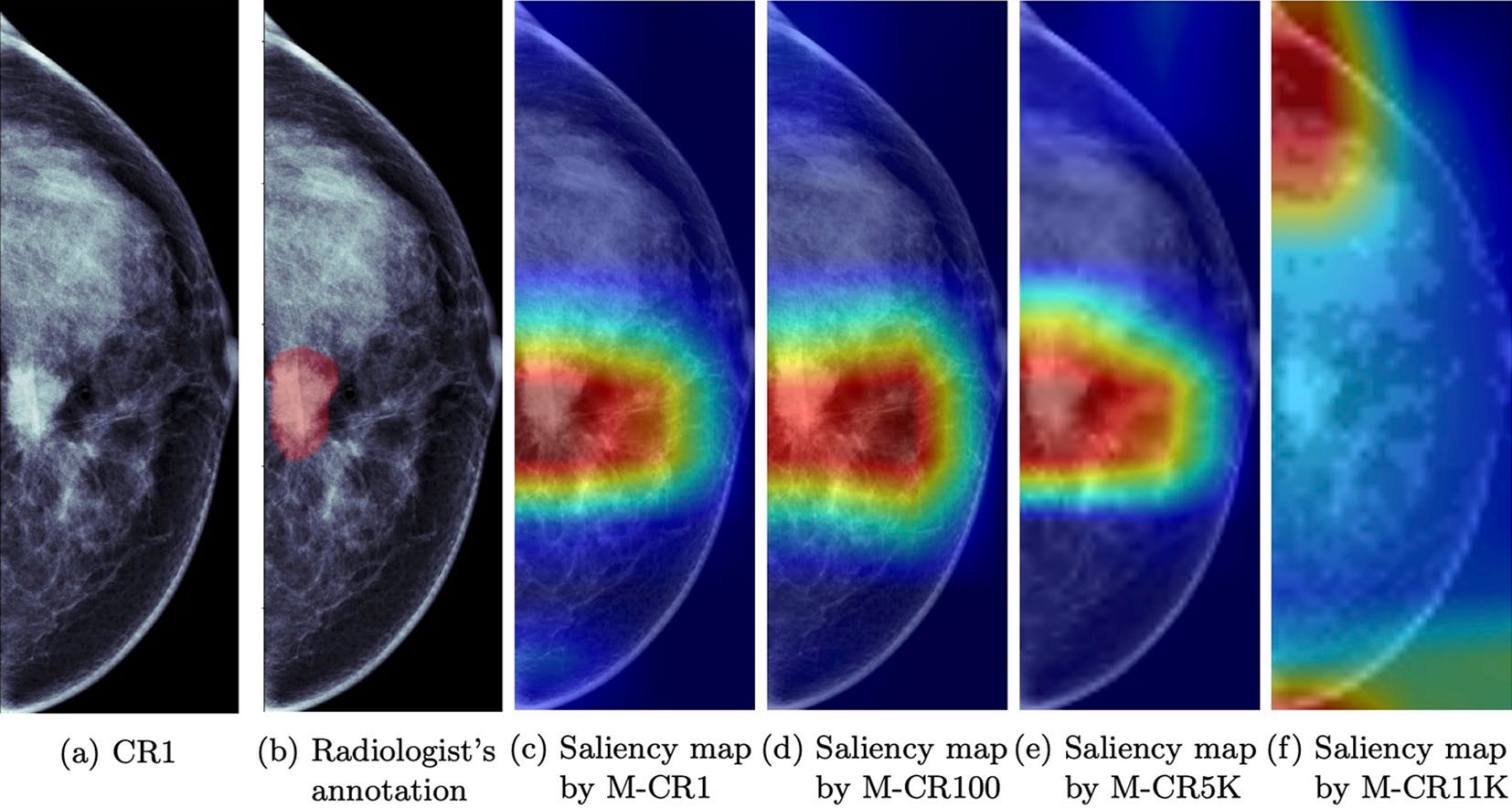
Saliency map results for a left craniocaudal view of a “malignant” case. Subfigure (**a**): the original image with a CR of one. Subfigure (**b**): the radiologist’s annotation on the original mammogram (i.e., ground truth). Subfigures (**c**)∼(**f**): saliency maps from models trained on images with the same compression ratio on images with CRs of 1, 100, 5 K, and 11 K. The areas marked in red are areas significant for prediction for each model.

To investigate the effects of image compression on the decision-making ability of our models, we used gradient-weighted class activation mappings to generate saliency maps^18^ for our CNN3 models. Figure 3 presents the original image of a malignant case in the LCC view and the respective saliency maps. The two leftmost images are both the original mammogram (i.e., CR1) with and without the radiologist’s annotation (i.e., ground truth), respectively. The remaining four images are the saliency maps on the images generated by their corresponding models.

In these saliency maps, the red signifies the area on the image with the highest influence on the decision of the respective network. The saliency maps of the three models (i.e., M-CR1, M-CR100, and M-CR5K) with similar classification performance display very similar distributions that encapsulate the ground truth. For this specific example, the models were able to accurately classify the mammogram as malignant with 100%, 99%, and 96% confidence for the CR1, CR100, and CR5K cases, respectively. In contrast, M-CR11K assigns a probability of 33% of the case being malignant, thereby inaccurately classifying it as normal or benign. Additionally, its associated saliency map completely misses the ground truth.

### Performance evaluation on the effect of data augmentation using image compression

Given that models are being trained and evaluated on more diverse populations^12^, and data storage guidelines regarding image compression vary across hospitals and countries^6^, we investigated the impact of testing mammogram classifiers using data compressed with a mix of compression ratios. Table 2 summarizes the classification performance of models trained on a single compression ratio evaluated on images with all eleven CRs. For both tables, the rows and columns represent models trained and test datasets with corresponding CRs, respectively, and the values represent the results for AUROC with its 95% CI in (a) and AUPRC with its 95% CI in (b), respectively. The classification performance of models from M-CR1 to M-CR5K remains consistent for test datasets compressed with CRs in the same range as their training datasets but decreases when evaluated on datasets with CRs of 10 K and 11 K. Interestingly, M-CR10K and M-CR11K improve performance on test datasets with relatively lower CRs, rather than those with CRs equal to those of their training datasets. These trends can be observed for both the AUROC and AUPRC.

**Table 2:** Classification performance of models trained and tested on images with different compression ratios.

Model/Data	CR1	CR15	CR20	CR25	CR50	CR100	CR500	CR1K	CR5K	CR10K	CR11K
**(a) AUROC with 95% confidence interval**											
M-CR1	–	0.87 (0.85, 0.89)	0.87 (0.85, 0.89)	0.87 (0.85, 0.89)	0.87 (0.85, 0.89)	0.87 (0.85, 0.89)	0.87 (0.85, 0.89)	0.87 (0.85, 0.89)	0.84 (0.82, 0.87)	0.73 (0.69, 0.76)	0.71 (0.68, 0.74)
M-CR15	0.87 (0.85, 0.89)	–	0.87 (0.85, 0.89)	0.87 (0.85, 0.89)	0.87 (0.85, 0.89)	0.87 (0.85, 0.89)	0.87 (0.85, 0.9)	0.87 (0.85, 0.89)	0.85 (0.82, 0.87)	0.73 (0.7, 0.76)	0.72 (0.69, 0.75)
M-CR20	0.87 (0.85, 0.89)	0.87 (0.85, 0.89)	–	0.87 (0.85, 0.89)	0.87 (0.85, 0.89)	0.87 (0.85, 0.89)	0.87 (0.85, 0.89)	0.87 (0.85, 0.89)	0.84 (0.81, 0.86)	0.72 (0.69, 0.75)	0.70 (0.66, 0.73)
M-CR25	0.86 (0.84, 0.89)	0.86 (0.84, 0.89)	0.86 (0.84, 0.89)	–	0.86 (0.84, 0.89)	0.86 (0.84, 0.89)	0.86 (0.84, 0.89)	0.86 (0.84, 0.89)	0.85 (0.82, 0.87)	0.73 (0.71, 0.76)	0.71 (0.69, 0.74)
M-CR50	0.87 (0.85, 0.9)	0.87 (0.85, 0.9)	0.87 (0.85, 0.9)	0.87 (0.85, 0.9)	–	0.87 (0.85, 0.9)	0.87 (0.85, 0.9)	0.87 (0.85, 0.9)	0.85 (0.83, 0.88)	0.73 (0.7, 0.76)	0.71 (0.68, 0.74)
M-CR100	0.88 (0.85, 0.9)	0.88 (0.85, 0.9)	0.88(0.85, 0.9)	0.88 (0.85, 0.9)	0.88 (0.85, 0.9)	–	0.88 (0.85, 0.9)	0.87 (0.85, 0.9)	0.84 (0.81, 0.86)	0.71 (0.67, 0.74)	0.70 (0.66, 0.73)
M-CR500	0.87 (0.85, 0.89)	0.87 (0.85, 0.89)	0.87 (0.85, 0.89)	0.87 (0.85, 0.89)	0.87 (0.85, 0.89)	0.87(0.85, 0.89)	–	0.87 (0.85, 0.9)	0.86 (0.83, 0.88)	0.73 (0.7, 0.76)	0.72 (0.69, 0.75)
M-CR1K	0.86 (0.83, 0.88)	0.86 (0.83, 0.88)	0.86 (0.83, 0.88)	0.86 (0.83, 0.88)	0.86 (0.83, 0.88)	0.86 (0.83, 0.88)	0.86 (0.84, 0.88)	–	0.84 (0.81, 0.87)	0.73 (0.7, 0.76)	0.72 (0.69, 0.75)
M-CR5K	0.87 (0.84, 0.89)	0.87 (0.84, 0.89)	0.87 (0.84, 0.89)	0.87 (0.84, 0.89)	0.87 (0.84, 0.89)	0.87 (0.84, 0.89)	0.87 (0.84, 0.89)	0.87 (0.85, 0.89)	–	0.78 (0.76, 0.81)	0.76 (0.73, 0.79)
M-CR10K	0.82 (0.78, 0.84)	0.82 (0.78, 0.84)	0.82 (0.78, 0.84)	0.82 (0.78, 0.84)	0.82 (0.78, 0.84)	0.82 (0.78, 0.84)	0.82 (0.79, 0.84)	0.82 (0.79, 0.84)	0.83 (0.79, 0.85)	–	0.79 (0.76, 0.82)
M-CR11K	0.80 (0.76, 0.82)	0.80 (0.77, 0.82)	0.80 (0.77, 0.82)	0.80 (0.76, 0.82)	0.80 (0.77, 0.82)	0.80 (0.77, 0.82)	0.80 (0.77, 0.82)	0.80 (0.77, 0.82)	0.80 (0.77, 0.83)	0.79 (0.76, 0.81)	–
**(b) AUPRC with 95% confidence interval**											
M-CR1	–	0.75 (0.7, 0.78)	0.75 (0.7, 0.78)	0.75 (0.7, 0.78)	0.75 (0.7, 0.78)	0.75 (0.7, 0.78)	0.75 (0.7, 0.78)	0.75 (0.7, 0.78)	0.68 (0.63, 0.72)	0.40 (0.35, 0.46)	0.38 (0.33, 0.43)
M-CR15	0.76 (0.71, 0.79)	–	0.76 (0.71, 0.79)	0.76 (0.71, 0.79)	0.76 (0.71, 0.79)	0.76 (0.71, 0.79)	0.76 (0.71, 0.79)	0.76 (0.71, 0.79)	0.70 (0.65, 0.74)	0.41 (0.36, 0.46)	0.39 (0.34, 0.44)
M-CR20	0.75 (0.7, 0.78)	0.75 (0.7, 0.78)	–	0.75 (0.7, 0.78)	0.75 (0.7, 0.78)	0.75 (0.7, 0.78)	0.75 (0.7, 0.78)	0.75 (0.7, 0.78)	0.67 (0.62, 0.71)	0.41 (0.36, 0.46)	0.38 (0.33, 0.43)
M-CR25	0.75 (0.7, 0.78)	0.75 (0.7, 0.78)	0.75 (0.7, 0.78)	–	0.75 (0.7, 0.78)	0.75 (0.7, 0.78)	0.75 (0.7, 0.78)	0.75 (0.7, 0.78)	0.70 (0.65, 0.74)	0.42 (0.37, 0.47)	0.39 (0.34, 0.44)
M-CR50	0.76 (0.71, 0.79)	0.76 (0.71, 0.79)	0.76 (0.71, 0.79)	0.76 (0.71, 0.79)	–	0.76 (0.71, 0.79)	0.76 (0.71, 0.79)	0.76 (0.71, 0.79)	0.70 (0.65, 0.74)	0.41 (0.36, 0.46)	0.38 (0.33, 0.43)
M-CR100	0.76 (0.71, 0.79)	0.76 (0.71, 0.79)	0.76 (0.71, 0.79)	0.76 (0.71, 0.79)	0.76 (0.71, 0.79)	–	0.76 (0.71, 0.79)	0.76 (0.71, 0.79)	0.68 (0.62, 0.72)	0.39 (0.34, 0.44)	0.37 (0.32, 0.42)
M-CR500	0.76 (0.71, 0.79)	0.76 (0.71, 0.79)	0.76 (0.71, 0.79)	0.76 (0.71, 0.79)	0.76 (0.71, 0.79)	0.76 (0.71, 0.79)	–	0.76 (0.71, 0.79)	0.70 (0.65, 0.74)	0.40 (0.35, 0.46)	0.37 (0.32, 0.42)
M-CR1K	0.74 (0.68, 0.76)	0.74 (0.68, 0.76)	0.74 (0.68, 0.77)	0.74 (0.68, 0.76)	0.74 (0.68, 0.76)	0.74 (0.68, 0.77)	0.74 (0.68, 0.77)	–	0.69 (0.64, 0.73)	0.41 (0.36, 0.46)	0.39 (0.34, 0.44)
M-CR5K	0.74 (0.69, 0.78)	0.74 (0.69, 0.78)	0.74 (0.69, 0.78)	0.74 (0.69, 0.78)	0.74 (0.69, 0.78)	0.74 (0.69, 0.78)	0.74 (0.7, 0.78)	0.75 (0.7, 0.78)	–	0.49 (0.43, 0.55)	0.44 (0.38, 0.49)
M-CR10K	0.66 (0.59, 0.69)	0.66 (0.59, 0.69)	0.66 (0.59, 0.69)	0.66 (0.59, 0.69)	0.66 (0.59, 0.69)	0.66 (0.59, 0.69)	0.66 (0.59, 0.69)	0.66 (0.59, 0.69)	0.65 (0.58, 0.69)	–	0.48 (0.42, 0.53)
M-CR11K	0.59 (0.53, 0.63)	0.59 (0.53, 0.63)	0.59 (0.53, 0.63)	0.59 (0.53, 0.63)	0.59 (0.53, 0.63)	0.59 (0.53, 0.63)	0.59 (0.53, 0.63)	0.59 (0.53, 0.63)	0.57 (0.51, 0.62)	0.47 (0.42, 0.53)	–

For each table, the row “M-CR15” denotes classification performance for a model trained on images compressed with compression ratio 15, while each cell is the relevant performance metric and 95% confidence interval for data when tested on data compressed with the CR denoted in the column header.

**Figure 4:**
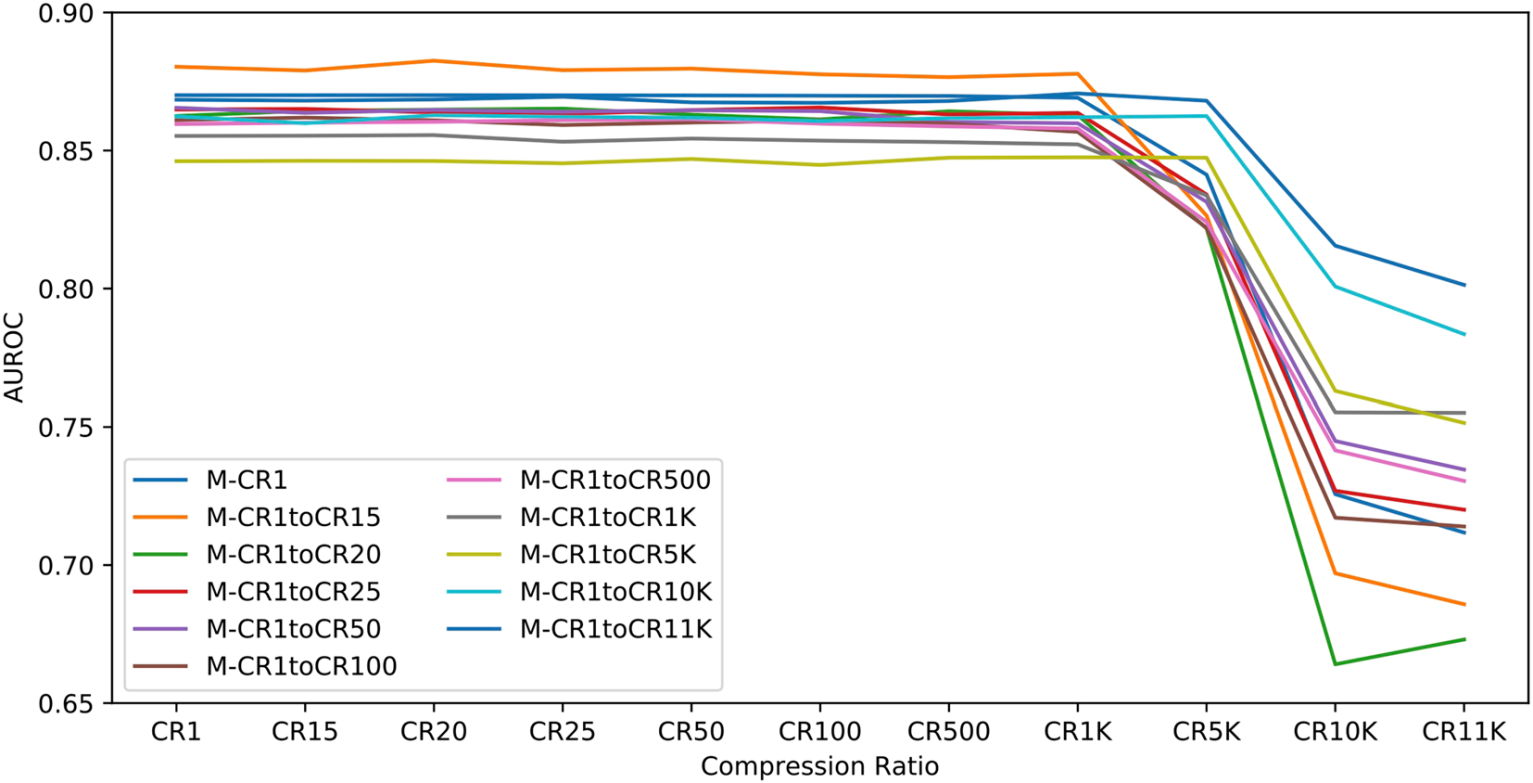
Performance of models trained with compression-based data augmentation. The plot shows AUROC scores of models trained using different CR ranges. Each label in the legend indicates a model trained on data with a mixture of CRs. For example, M-CR1toCR11K refers to the model trained on a dataset with a mixture images with different CRs ranging between 1 and 11 K. Each model is evaluated on images with a single CR of 1, 15, 20, 25, 50, 100, 500, 1 K, 5 K, 10 K, and 11 K, respectively.

Figure 4 demonstrates the change in performance of the data augmentation due to image compression. We augmented the dataset with compression ratios by compressing all images in the training set with a range of compression ratios. For example, M-CR-1to15 is the model trained on both the original, uncompressed data and the same data compressed with CR 15. Models trained on a mixed set of images, with CRs between 1 and 11 K, achieves higher performance on images with CRs of both 10 K and 11 K than models trained on images solely with a CR of 1. This indicates that the use of compression for data augmentation improves model generalization when tested on different compression ratios.

## Discussion

This study investigated the effects of image compression on the performance of deep learning (DL)-based mammogram classification. As previous work already investigated the impact of image compression on human classification performance^7–9^ and because DL methods are most commonly benchmarked against other DL models, our work focuses on deep learning-based methods. We compressed mammograms using various compression ratios (CRs) using JPEG 2000 compression. We then trained and evaluated models based on convolutional neural networks to classify each paired view as either normal/benign or malignant with the compressed images. The results show that models trained and tested on images compressed using a single CR less than or equal to 5 K have an area under the receiver operating characteristic curve (AUROC) of 0.86–0.88 (± 0.01–0.02, 95% confidence interval (CI): 0.85 to 0.89) and an area under the precision-recall curve (AUPRC) of 0.74–0.76 (± 0.02–0.04, 95% CI 0.68 to 0.79). In contrast, models trained and tested on images with CRs of 10 K and 11 K had an AUROC of 0.81 (± 0.02, 95% CI 0.78 to 0.83) and 0.78 (± 0.03, 95% CI 0.75 to 0.81) and AUPRC of 0.52 (± 0.06, 95% CI 0.46 to 0.57) and 0.46 (± 0.05, 95% CI 0.40 to 0.51), respectively. When we generated saliency maps to visualize the areas in each image the given model views as significant, models trained on mammograms with CRs less than or equal to 5 K had maps that encapsulated a radiologist’s label for malignant cases. However, models trained on images with CRs greater than 5 K (i.e., CR of 10 K and 11 K) had saliency maps that failed to encapsulate the ground truth label. In addition, we explored the effect of having different CRs for training and testing the classification performance. Models trained on a mixture of images with CRs ranging from 1 to 11 K achieve a higher performance when tested on images with CR of 10 K or 11 K than a model trained on uncompressed images.

DL models for mammograms have been applied to a range of clinical tasks in the past two years such as the breast density classification, detecting lesions in mammograms, and breast cancer risk prediction^10–15,19^. With the success of these models, there has been increasing interest in cross-hospital as well as international collaborations in this area. This includes a recent study where models were trained on mammograms from the U.S. and tested on data from Sweden^19^, as well as a study where models were trained and tested on a mix of mammograms from the U.S. and the U.K. or trained solely on U.K. data and tested on U.S. data^12^. However, despite the diversity of compression methods, hospital policies, and healthcare law regarding image compression, the prior work on DL related to mammography does not incorporate compression as a factor in their models. Previous work on machine learning applications to clinical imaging focused on the impact of image compression on histographical classification^20,21^, data loss caused by lower image resolution on various medical images^22,23^, and the use of deep learning to apply image compression to mammograms^24,25^. However, we believe that our study is the first to address image compression as a factor in DL classification in radiology. Our study shows that an appropriate amount of image compression is able to help address the overhead associated with image storage and transmission without impacting classification performance for DL-based models downstream.

Our study comes with many limitations. Although all mammograms used in this study were obtained using four devices from two different vendors, they were drawn from a single cancer center in the Republic of Korea. Additionally, we only evaluated the impact of image compression on binary classification of mammograms; future studies may investigate other salient tasks such as BI-RADS classification, object detection or instance segmentation.

In conclusion, we investigated an issue unaddressed in previous studies, by evaluating the impact of image compression on the performance of DL-based models when tested on images with various CRs. With the exception of extreme cases (e.g., images compressed with CRs greater than 5 K), our results show that training and testing models on a single CR does not impact classification performance. In future work, we will examine the impact of image compression on other tasks, such as object detection and semantic segmentation, and evaluate the classification performance on other types medical images.

## Materials and methods

### Study design

For this retrospective study, we collected clinical information and full-field digital mammograms from 48,871 unique subjects, screened at the National Cancer Center in the Republic of Korea between January 1, 2013 and December 31, 2018. This study was approved by the institutional review board of the National Cancer Center-Korea, with a waiver for written informed consent (2019–0126). In addition, we confirm that all methods were performed in accordance with the relevant guidelines and regulations.

### Image acquisition and compression

All images used in this study were sourced from a single clinical center to ensure that diagnoses were confirmed using pathology and patient outcomes. Each DICOM file was originally compressed using JPEG 2000 lossless compression, and all pixel values were decompressed before processing. The 9111 full-field digital mammograms used in this study were obtained using four devices from two different vendors (Lorad Selenia and Selenia Dimensions from Hologic and Senograph 2000 and Senograph DS from GE Medical Systems). Each study includes four paired views: left mediolateral oblique (LMLO), right mediolateral oblique (RMLO), left craniocaudal (LCC), and right craniocaudal (RCC). The image dimensions for each view were between 2294 and 4096 pixels along the x-axis and between 1914 and 3328 pixels along the y-axis.

**Figure 5:**
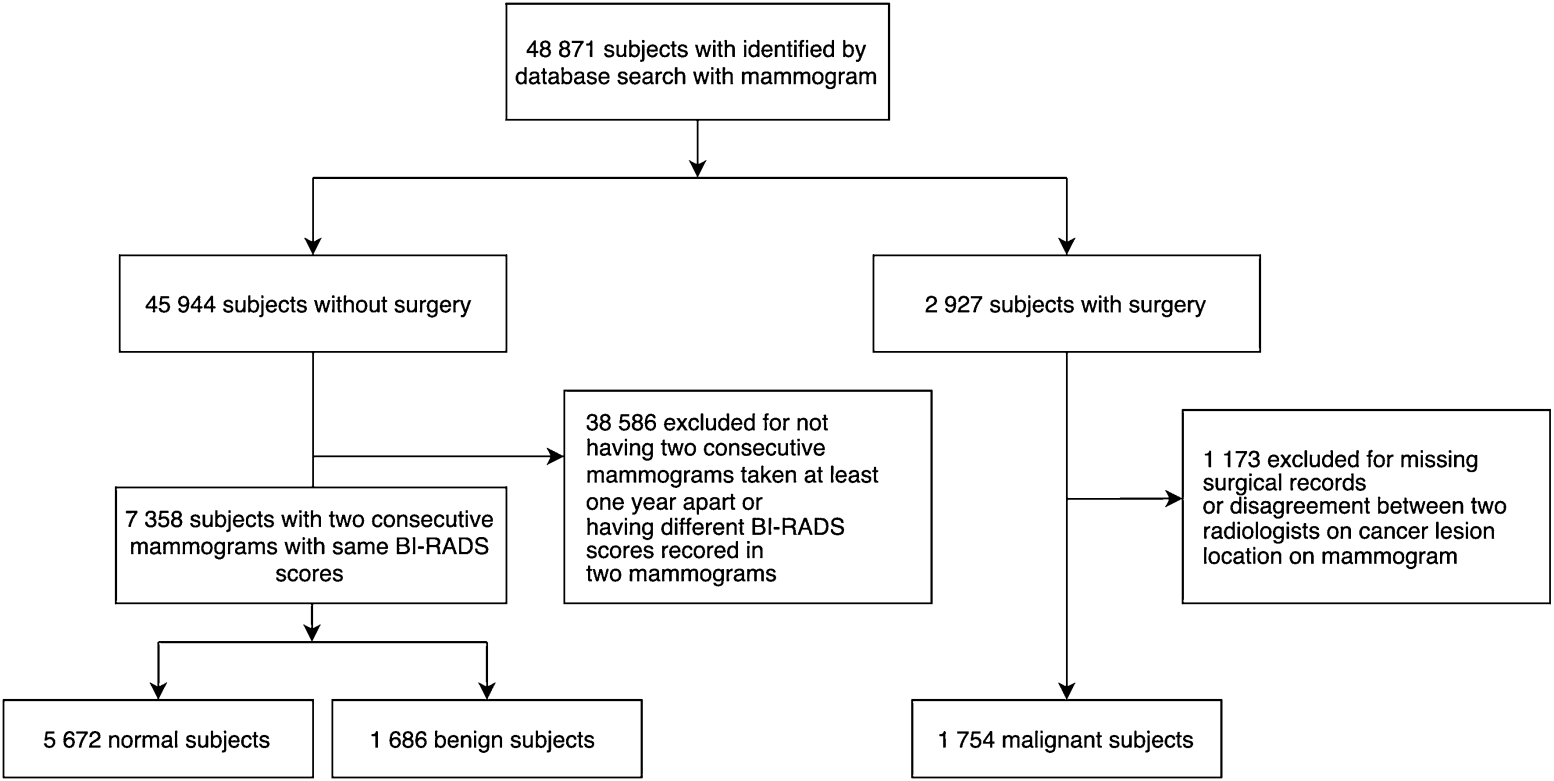
Subject selection flowchart.

According to the selection criteria, only subjects with cancer lesions deemed normal/benign who have had two consecutive mammograms taken at least one year apart, with the mammograms being assigned the same breast imaging reporting and data system (BI-RADS) score, have been included in this study. Cancer lesions deemed normal were assigned a BI-RADS score of one, while those deemed benign were assigned scores of two and three. Only the most recent mammogram of a qualifying subject was included in our study. Subjects with malignant cancers underwent surgery, and the cancer lesion from their preoperative mammograms was diagnosed independently by two radiologists. Of the 9111 unique subjects, our study included 5672 subjects with cancers deemed normal, 1686 benign, and 1754 malignant as shown in Fig. 5. Table 3 lists the number of subjects grouped by their characteristics, including BI-RADS score, lesion position, and TN stage.

**Table 3:** Subject characteristics.

	Normal	Benign	Malignant
Number of subjects	5672	1686	1753
**Age**			
< 40	22 (0.4%, 35.31 ± 3.04)	50 (3.0, 35.6 ± 3.89)	144 (8.2%, 36.27 ± 2.92)
40–49	1217 (21.5%, 44.80 ± 2.76)	475 (28.2%, 45.19 ± 2.65)	607 (34.6%, 45.17 ± 2.76)
50–59	2175 (38.3%, 54.33 ± 2.79)	735 (43.6% 54.34 ± 2.70)	594 (33.9%, 54.14 ± 2.94)
60–69	1569 (27.7%, 63.62 ± 2.81	327 (19.4%, 63.38 ± 2.91)	279 (15.9%, 63.78 ± 2.69)
70–79	640 (11.3%, 72.76 ± 2.50)	97 (5.8%, 72.84 ± 2.22)	110 (6.3%, 73.56 ± 2.74)
80 >	49 (0.9%, 81.55 ± 2.61)	2 (0.1%, 80.00 ± 0.00)	19 (1.1%, 82.05 ± 1.54)
**Lesion position**			
Left	–	609 (36.1%)	886 (50.5%)
Right	–	524 (31.1%)	857 (48.9%)
Both	5672 (100%)	553 (32.8%)	10 (0.6%)
**T stage**			
1	–	–	1083 (61.8%)
2	–	–	614 (35.0%)
3	–	–	31 (1.8%)
4	–	–	2 (0.1%)
Etc	–	–	23 (1.3%)
**N stage**			
0	–	–	1234 (70.4%)
1	–	–	399 (22.8%)
2	–	–	61 (3.5%)
3	–	–	13 (0.7%)
Etc	–	–	2 (0.1%)

Age denotes the number of subjects and a proportion, average and standard deviation in the parentheses, and others only presents proportions.

JPEG and JPEG 2000 compression are the two most common image compression methods used in medical imaging^5^. For this study, we selected JPEG 2000 compression as it allows for a wider dynamic range of 16-bits, in contrast to the 8-bit range of JPEG compression. The compression ratio (CR) achieved by these methods is defined as the file size of the uncompressed image (i.e., the original image) divided by that of its compressed image (CR = original file size/compressed file size). This metric is often represented as a single number; for example, a 30 MB image compressed to approximately 3 MB has a CR of 10. A higher CR implies more compression, which can possibly lead to data loss and may introduce visual artifacts in the compressed image.

### Model development and evaluation

This study aims to classify a mammogram as either normal/benign or malignant using a DL-based model. We first split the mammograms into two groups: 7358 as normal/benign and 1753 as malignant (approximately a 4:1 ratio). JPEG 2000 compression is then applied to each of the four views of a given mammogram. Next, each compressed view is resized to 30% of its original image size and then cropped. On the x-axis, all pixels in the right half and the LMLO and LCC views, left half of the RMLO and RCC views are cropped. For the y-axis, pixels in the top fifth of the image in the LMLO and RMLO views and the bottom fifth of the image for the LCC and RCC views are cropped. We then concatenate the two views from each side into a single input image (i.e., combining the LMLO and LCC views from the left breast or the RMLO and RCC views from the right breast).

Each convolution layer has a 6 × 6 filter, stride of 2, and padding of 2. After the convolution, batch normalization (BatchNorm)^26^, Rectified Linear Unit (ReLU) activation^27^, and max pooling are applied. Each of the two fully connected layers have BatchNorm, a ReLU activation, and max pooling applied to the respective input. The CNN models were trained using binary cross-entropy for the loss function and optimized using the Adam optimizer^28^ with learning rate of, of $0.5$, $\upbeta _{1} = 0.9$, $\upbeta _{2} = 0.999$, and $\epsilon = 10^{ - 8}$. All models were built using Ubuntu (version 18.04), Python (version 3.6), and PyTorch (version 1.2). The model classifies a mammogram as malignant if a malignant lesion is suspected with a high probability in any view of the concatenated input image. The work was performed on a workstation equipped with an Intel Xeon Silver 4114 processor, 128 GB RAM, and two NVIDIA RTX TITAN GPUs. We split the images into training, validation, and test datasets, with a 7:1:2 ratio.

We conducted the following two experiments: (1) a performance evaluation of models trained on different CRs and (2) a performance evaluation of image compression as a means of data augmentation.

The first experiment demonstrates the impact of compression on the classification performance of the models. Each model is evaluated with the receiver operator characteristic curve (ROC) curve and precision recall (PR) curve. We highlighted the important regions in an image for the model prediction using saliency maps generated by gradient-weighted class activation mappings^18^ and qualitatively analyzed the classification performance of the models trained with different CRs.

The second experiment is motivated by the scenario of multi-hospital collaborations where participating medical centers may have different policies regarding image compression^13^. First, we demonstrate that the classification performance drops when models are trained and tested on images compressed with different CRs. We then demonstrate an increase in the robustness of the models when image compression is used for data augmentation.

We reported metrics from five-fold cross validation for both experiments.

## Data Availability

The dataset is sourced from the National Cancer Center in the Republic of Korea. All subject information was deidentified and can be accessed through AIHUB (http://www.aihub.or.kr/aidata/134), a platform providing infrastructure for AI technique and service development. All code used in this study is through the National Cancer Center Healthcare AI Team GitHub account (https://github.com/nccaiteam) after publication.
